# Byproducts of Sesame Oil Extraction: Composition, Function, and Comprehensive Utilization

**DOI:** 10.3390/foods12122383

**Published:** 2023-06-15

**Authors:** Yuan Wan, Qiaoyun Zhou, Mengge Zhao, Tao Hou

**Affiliations:** 1 College of Food Science and Technology, Huazhong Agricultural University, Wuhan 430070, China; wanyuan410@webmail.hzau.edu.cn (Y.W.); zhouqiaoyun@webmail.hzau.edu.cn (Q.Z.); zhaomenggehzau@163.com (M.Z.); 2 Shenzhen Institute of Nutrition and Health, Huazhong Agricultural University, Shenzhen 518120, China; 3 Shenzhen Branch, Guangdong Laboratory for Lingnan Modern Agriculture, Genome Analysis Laboratory of the Ministry of Agriculture, Agricultural Genomics Institute at Shenzhen, Chinese Academy of Agricultural Sciences, Shenzhen 518000, China

**Keywords:** sesame meal, sesame lignans, functional activities, comprehensive utilizations

## Abstract

Sesame is principally used to generate oil, which is produced by chemical refining or pressing. Sesame meal, as a main byproduct of sesame oil extraction, is usually discarded, causing resource waste and economic loss. Sesame meal is rich in sesame protein and three types of sesame lignans (sesamin, sesamolin, and sesamol). Sesame protein extracted via a physical method and an enzymic method has balanced amino acid composition and is an important protein source, and thus it is often added to animal feed and used as a human dietary supplement. Extracted sesame lignan exhibits multiple biological activities such as antihypertensive, anticancer, and cholesterol-lowering activities, and therefore it is used to improve the oxidative stability of oils. This review summarizes the extraction methods, functional activities, and comprehensive utilization of four active substances (sesame protein, sesamin, sesamolin, and sesamol) in sesame meal with the aim to provide theoretical guidance for the maximum utilization of sesame meal.

## 1. Introduction

Sesame (*Sesamum indicum* L.) is an ancient oilseed crop with high contents of lipids (45–55%) and proteins (20–25%) that is beneficial to humans’ health. It is widely distributed all over the world and, traditionally, India, Sudan, Myanmar, China, and Tanzania are the main producers of sesame. According to the statistics of the Food and Agriculture Organization of the United Nations in 2020, the global sesame cultivation area was 13.9 million hectares with a yield of 6.80 million tons (http://www.fao.org/faostat/en/#data/QC/, accessed on 9 August 2021). Since its oil content is approximately 45–50% [1], sesame is used primarily for extracting oils and preparing milk-like beverages [2]. It is also used extensively for producing confectionery, bakery products, and sesame butter. Sesame oil is one of the most popular flavored essential oils, and about 0.4 million tons of flavored sesame oil are consumed every year [3]. However, after oil extraction, a large amount of sesame meal is discarded, causing huge waste.

Sesame meal is a protein-rich byproduct which is currently discarded or used as animal feed. Actually, it can be used as an important economically viable protein-rich food source to revitalize oilseed cultivation [4]. Sesame meal contains about 50% plant protein and is rich in methionine and tryptophan, which distinguishes it from other oil seeds [5]. Plant proteins provide essential amino acids for the human body and possess various desirable characteristics. Therefore, sesame meal has high application prospects as a protein source or raw material in the food industry. It can be used not only in the preparation of cattle, pigs, and poultry feed, but also as a dietary ingredient of fish feed. Additionally, the byproducts of sesame oil also contain various kinds of other bioactive substances such as sesamin, sesamolin, sesamol, and starch [6].

**Figure 1:**
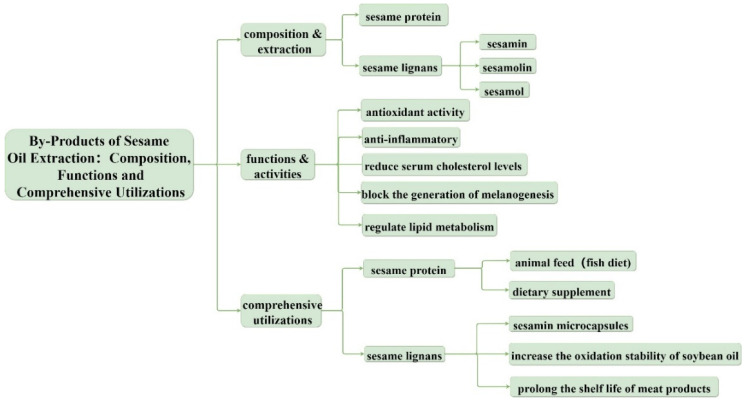
Framework schematic. Byproducts of sesame oil extraction: composition, function, and comprehensive utilization.

Although the demand for sesame oil, sesame paste, and other sesame products is increasing, sesame meal has not been fully utilized in our daily life. With the advancement in sesame meal processing technology, the nutritional values of sesame meal have been improved. Thus, in the present study, we aim to review the research progress and utilization status of bioactive substances in sesame byproducts (Figure 1) in order to provide theoretical guidance for their future comprehensive utilization. This review will be of great significance to the production, marketing, and international trade of sesame processing byproducts, and it can better guide and optimize feed production.

## 2. Composition and Extraction of Bioactive Substances in Sesame Meal

### 2.1. Sesame Protein

Sesame protein is the most important bioactive substance in sesame oil byproducts, most of which are composed of globulins (67.3%), albumins (8.6%), prolamines (1.4%) and glutens (6.9%) [7]. At present, sesame protein has become a favorable protein source for human beings. Protein extraction from sesame residue and comprehensive utilization can increase the sesame byproduct utilization rate, exhibiting broad application prospects.

#### Extraction of Sesame Protein

In accordance with Osborne sequential extraction and different solubility, sesame protein has been classified into four types, namely, water-soluble albumins, salt-soluble globulins, prolamins soluble in alcohol/water mixtures, and glutens soluble in dilute acid or alkali [8]. According to the characteristics of different types of proteins, different extraction methods are adopted.

Sesame protein mainly contains alkali-soluble protein, which has a high solubility under alkaline conditions, and thus preparation methods of sesame protein are mainly alkali extraction followed by acid precipitation. The extraction yield of sesame protein obtained via this method is about 67% [9]. Nevertheless, at a high pH (10.0), the extraction efficiency is lowered with the decrease in the solubility of proteins. Alkali extraction has the advantages of simple operation, easy control, and low cost. However, strong alkali tends to produce lysyl alanine and other toxic substances, cause nutrient loss, and enhance the Maillard reaction, thus causing the denaturation and hydrolysis of proteins and adversely affecting the color of the products, eventually resulting in the decline in the commercial quality of byproducts [10]. In order to improve the traditional extraction technology of sesame protein using alkali solution and acid isolation, the preparation technology of sesame protein from subcritical sesame meal has been optimized [11]. The purity and recovery rate of the protein extracted from the subcritical sesame meal are superior to those from the high-temperature sesame meal because the polyphenol enzymes in subcritical sesame are not inactivated [12]. Subcritical water is used to hydrolyze rice bran and sesame residue into more valuable proteins and amino acids.

Apart from alkali-soluble acid precipitation process, ultrafiltration and ultrasound are available for assisting sesame protein extraction. Ultrafiltration is a kind of molecular membrane separation technology developed in recent years. It is characterized by low energy consumption, no phase transition, and high efficiency [13]. Ultrafiltration has been widely used in water treatment, the chemical industry, food, pharmaceutics, environmental protection, and bioengineering, and it has become one of the most effective methods to enrich protein [14]. One of the most important characteristics of protein products obtained via ultrafiltration is the good solubility. One previous study investigated the properties of sesame protein and prepared sesame protein using multiple methods such as alkali extraction + salting out, alkali extraction + ultrafiltration + acid precipitation, alkali extraction + ultrafiltration + salting out, and subcritical extraction from defatted sesame meal. Results showed that alkali extraction + ultrafiltration + salting out was the most suitable method for sesame protein preparation, and the yield and purity of products obtained via this method were higher than those obtained using other methods, which were 75.47% and 83.18%, respectively [15]. In addition, ultrasound is a new method for producing proteins. Compared to other technologies, ultrasound is less energy consuming and more time saving. Sun et al. (2017) used ultrasound, homogenate, and microwave technologies to assist in the alkali extraction of plant-source proteins, and found that the ultrasound-assisted alkaline method had the highest protein extraction rate. Certainly, it is worth mentioning that the solubility, water absorption, emulsification activity, and foaming stability of the proteins vary with physical techniques. Moreover, the number of sulfhydryl groups and disulfide bonds is also significantly affected by physical processing technologies. More hydrophobic groups are exposed in the process of ultrasound-assisted sesame protein extraction, thus leading to the increase in surface hydrophobicity [16].

Compared with standard alkaline extraction methods, the enzymatic extraction method presents multiple advantages such as increasing the protein yield, total phenol content, and antioxidant capacity [17]. The enzymatic extraction of protein involves breaking down proteases into peptides followed by extraction and separation. Proteolytic enzymes are selected to specifically cleave peptide bonds for protein enzymatic modification, which has been widely used in the food industry. Many factors affect the extraction rate of sesame protein, such as enzyme specificity, hydrolysis time, hydrolysis degree, and pretreatment methods. For example, the enzymolysis of the multi-enzyme complex is significantly higher than that of the corresponding single enzyme [18]. One previous study hydrolyzed sesame protein with alkaline protease and papain in single-enzyme hydrolysis, one-step double-enzyme hydrolysis, and stepwise double-enzyme hydrolysis [19], and found that stepwise double-enzyme hydrolysis was superior to single-enzyme hydrolysis and one-step double-enzyme hydrolysis. With the increase in enzymolysis time, the number of protease peptide bonds gradually decreases. The increase in enzymolysis efficiency tends to be slow, and the enzymolysis products continue to accumulate. At this time, the second enzyme was added to continue enzymolysis, which could continue enzymolysis according to its catalytic characteristics and cutting site, making the degree of hydrolysis rapidly increase again. The hydrolysis ability of alkaline protease is better than that of the same amount of papain [20]. Apart from the enzyme specificity, the pretreatment for proteins also affects the hydrolysis efficiency. The high recovery of sesame protein can be obtained by pretreating sesame meal; degreasing sesame with surfactant and enzymatic hydrolysis [21].

### 2.2. Sesame Lignans

#### 2.2.1. Sesamin, Sesamolin, and Sesamol

Lignans are a group of compounds consisting of dimers of phenylpropane units. Sesamin, sesamolin, and sesamol are the three main lignans in sesame, and they are also important plant antioxidant lignans. Sesame lignans have a variety of biological effects, exhibiting high activity in carbohydrate and lipid metabolism, hypertension, inflammation, and free radical scavenging [22].

**Figure 2:**
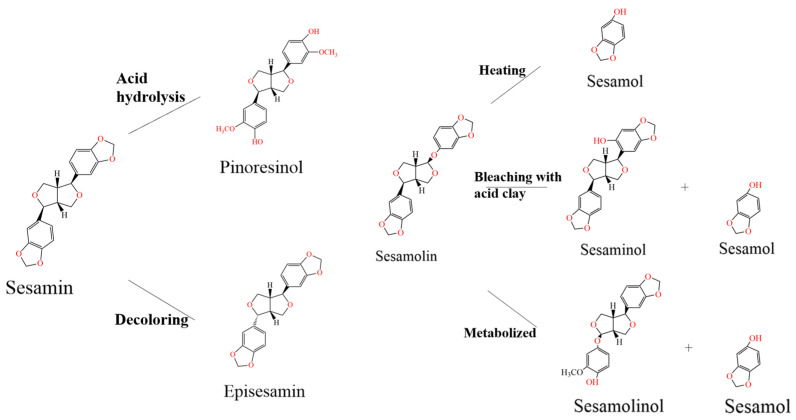
Schematic of interconversion process and conditions of lignans and compounds isolated from sesame. The major aglycon lignans are sesamin and sesamolin. The minor aglycon lignans of sesame oil include sesamol, sesaminol, sesamolinol, pinoresinol, and episesamin.

Sesamin and sesamolin were first isolated in the 1950s, and they are the major sesamin lignans. Sesamolin is a particular lignan compound with physiological activity in sesame oil. The average mass fraction of sesamolin in sesame oil is 0.27%, second only to sesamin (mean 0.36%) [23]. This compound has multiple pharmacological activities, such as antioxidant, antibacterial, neuroprotective, and anticancer activities. Sesamol, a thermally stable compound, is derived from sesamolin during oil processing (roasting and decolorization) under high temperature and moisture conditions (Figure 2). Sesamol is a major lignan isolated from sesame and sesame oil, displaying bioactive activities mainly including antioxidant, lipid-lowering, and anti-inflammatory activities [24].

#### 2.2.2. Extraction, Separation, and Detection of Sesame Lignans

The traditional extraction methods of sesamin from sesame oil include the organic solvent method and the stripping process. The former has its inevitable limitations, while the latter requires high energy consumption [25]. Macroporous adsorption resin is a new type of non-ionic organic polymer adsorbent, and its merits include large adsorption capacity, simple operation, environmental friendliness, and reusability. Therefore, it has become a desirable carrier for extracting and refining natural active ingredients [26]. Macroporous resin has been used as an adsorption surface to obtain sesamin crystals via the crystallization of desorption product [25]. In the past few years, magnetic solid phase extraction (MSPE) technology has attracted extensive attention in the field of sample preparation. An MSPE–high-performance liquid chromatography (HPLC) method based on graphene oxide has been established for the adsorption of target compounds from sesame oil by combining synthetic graphene oxide with hydroxylated Fe_3_O_4_ MNPs [27].

HPLC is the most commonly used detection method for sesamin in numerous studies. HPLC and gas chromatography–mass spectrometry (GS/MS) are very suitable for the determination of sesamin, asarin, and sesamolin in sesame oil samples according to the specified sensitivity requirements. The isolated lignan mixture is subjected to semi-preparative HPLC and thin-layer chromatography (TLC), resulting in the successful separation of putative sesamin from sesamolin as two independent fractions [28]. After passing through an alumina column and semi-preparative HPLC, two compounds from the unsaponifiable fraction of sesame oil are isolated. The separated compounds are analyzed by re-chromatography with purity exceeding 99%. GC-MS analysis confirms that these isolated compounds are sesamin and sesamolin. In order to eliminate the impact of oil components on oil samples, the pretreatment of oil samples is a necessary step in the detection of lignans in sesame oil. Solid-phase extraction, liquid–liquid extraction, TLC, and saponification are the prime pretreatment methods, and another pretreatment method is the further extraction of the sesame oil from the pressed sesame meal using supercritical CO_2_ extraction [29]. Furthermore, high-speed counter-current chromatography (HSCCC) is a preparative all-liquid chromatographic technique based on partitioning between two immiscible liquid phases. HSCCC has emerged as a superior technique for the preparative separation of natural compounds, and it has been commonly used to prepare and separate sesamolin from sesame oil.

However, conventional extraction and preparation methods such as chromatography (thin-layer chromatography, column chromatography, and, later, HPLC) have comparatively low sensitivity and specificity. Some studies have employed atmospheric pressure chemical ionization–mass spectrometry (APCI/MS) and electrospray–mass spectrometry (ECI/MS) for the detection of sesame lignans [30]. Following HPLC, CPC (centrifugal partition chromatography) has been used in the extraction of sesamin from defatted sesame because it can carry more sample loads and has a comprehensive range of solvent choices. CPC is a solid-free separation technology based on continuous liquid–liquid partitioning, and it is an effective tool for the separation and purification of components from natural products. CPC, a preparative separation method using consecutive sample injection, was developed to obtain sesamin and sesamolin from defatted sesame meal extracts. This method involves four repeated sample injections, thus obtaining highly pure sesamin and sesamolin from oil cake. CPC is also used to isolate lignans on both a semi-preparative and a preparative scale [31].

**Figure 3:**
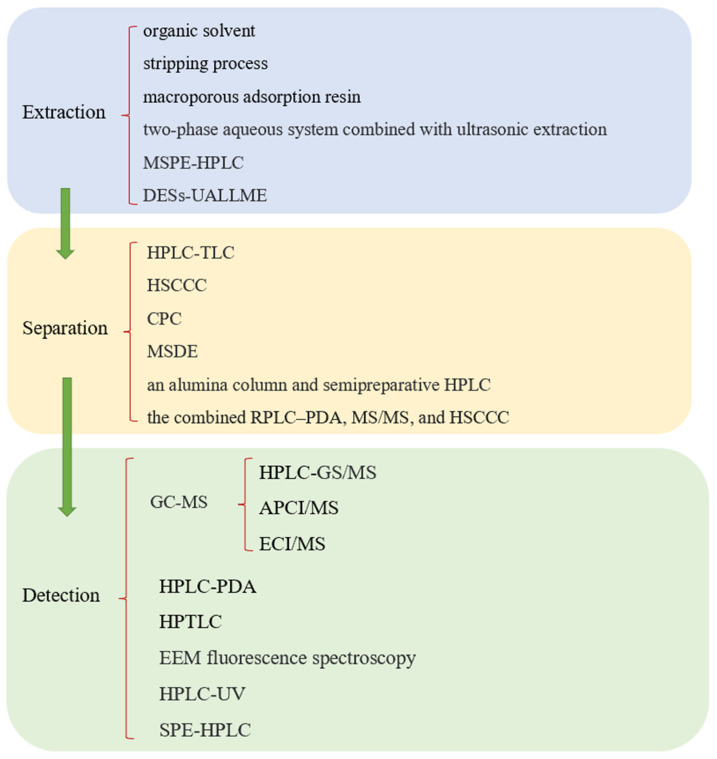
Schematic diagram of extraction, separation, and detection of sesame lignans.

With the development of modern technology, high-performance thin-layer chromatography (HPTLC) has merged as an effective, simple, and economical alternative to HPLC-PDA, and it is successfully applied for the quantification of sesamin and sesamolin in sesame samples (Figure 3). HPTLC provides good separation, it enables the visual inspection of the metabolites of interest, and it also allows for screening a large number of samples within a short time; thus, it is a valuable, rapid tool for quality control purposes [32]. However, sample pretreatment is time consuming and complex in operation, making chromatographic methods unsuitable for rapid or online analysis. Therefore, it is urgent to develop low-cost and rapid methods for the determination of sesamin and sesamolin in sesame oil samples. Excited emission matrix (EEM) fluorescence spectroscopy is a fast and non-destructive analytical technique. Under the condition of simple sample pretreatment, the combination of excited emission fluorescence spectroscopy and self-weighted alternating trilinear decomposition is a practical and sensitive method to determine the lignan content in sesame oil [33].

Additionally, the two-phase aqueous system combined with the ultrasonic extraction of lignans, reverse-phase liquid chromatography–photodiode array tandem mass spectrometry, and HSCCC have been used to determine and purify sesamin in sesame oil [34]. The efficient ultrasound-assisted liquid–liquid microextraction technology and microwave-assisted water vapor distillation–solvent extraction method [35] have been established for sesamin separation and the quantitative and qualitative determination of sesamin in sesame. After extracting the essential oil via steam distillation, the powder residue is relatively dry. Hence, this method can save energy consumption, and the loss of sesamin can be reduced during the subsequent extraction process and improve the essential oil yield.

The extraction method of sesamol is similar to that of sesamin and sesamolin to some degree. Recently, anion exchange solid-phase extraction (SPE) coupled with high-performance liquid chromatography (SPE-HPLC) [36] has been established for the determination of sesamol in sesame oil, and it is efficient, convenient, and accurate [37]. The SPE has the advantages of high recovery, less solvent consumption, and quick and convenient operation, and thus it is widely used in sample purification. The SPE can efficiently extract sesamol and remove matrix substances interfering with HPLC analysis. Sesamol is freely soluble in organic solvents such as chloroform, methanol, and ether [38], and it can also be extracted through organic solvents. In addition to these methods, ultrasound-assisted liquid–liquid microextraction (UALLME) has been used to extract sesamol from sesame oil based on deep eutectic solvent (DESs) [39]. GC-MS and HPLC are commonly used to detect sesamol.

## 3. Functions and Activities of Sesame Protein and Sesame Lignans

### 3.1. Sesame Proteins 

#### 3.1.1. Functional Properties of Sesame Proteins 

The functional properties of protein include solubility, foaming, gelling, and emulsifying properties. The total protein isolated from sesame has high solubility under acidic and alkaline conditions, and its emulsification and foaming properties are better than those of other proteins [40]. Sesame protein can add flavor to foams, lotions, and gels used in many foods and it has potential to be used in food formulation systems. The addition of sesame protein to wheat flour dough not only increases the protein content, the mineral content, and the total essential amino acids, but it also enhances various properties of the dough, such as its water absorption, expansion, and softening [41]. Therefore, there is increasing interest in improving the functional properties of sesame protein to ensure attractive products [42].

The extraction method of protein has a certain influence on its functional properties. The emulsifying foaming ability and emulsion stability of sesame protein obtained via ultrafiltration are remarkably higher than those obtained via isoelectric precipitation [13]. The solubility, emulsification, and foaming properties of sesame protein vary with pH and NaCl concentration. The foaming and emulsion stability of sesame protein decrease with the increasing hydrolysis degree [4]. The decrease in foam number and foam stability may be due to the increasing small and medium peptides in the hydrolysate. The high hydrolysis degree usually facilitates the diffusion of the peptide across the interface, and the production of hydrolysates with non-charged amino acid residues at the C-terminal of the peptide may account for the low emulsifying ability. The increase in ionic strength of dispersion media contributes to the increase in the foaming and emulsifying properties. For instance, ultrasound with a high-power (20 kHz) probe changes the functional properties of protein such as solubility and foaming ability by altering the temperature and conductivity of the medium around the protein [43]. The high-intensity ultrasound opens the hydrophilic parts of the amino acids toward the water, thus altering conformation and structure of the protein, eventually enhancing the solubility of the protein. The homogenization of ultrasound usually makes protein and fat particles disperse more evenly, thereby improving the foaming properties.

Pretreatments of sesame affect the functional properties of sesame protein. The protein content of germinated sesames increases slightly (about 10%) [44]. Germination treatment significantly increases the content of the free sulfhydryl group and the surface hydrophobicity of the sesame protein. After germination, the bands of high-molecular-weight protein gradually disappear and the secondary structure of the protein changes. The high-molecular-weight protein is decomposed into low-molecular-weight protein, thus improving the solubility and the emulsion stability of sesame protein [45]. Soaking sesame for 12–14 h prior to germination can be used to improve the composition and functional properties of sesame, thereby increasing the utilization rate of flour. Germination significantly increases the free sulfhydryl group number and surface hydrophobicity of sesame protein, indicating that the protein molecular structure is unfolded [46]. In addition to the germination treatment, the soaking and boiling treatments of sesame also significantly affect the functional characteristics of the flour. Foam is more stable for the raw meal than for the heat-processed meal [47]. 

#### 3.1.2. Biological Activities of Sesame Protein/Peptide 

Currently, research on the biological activities of sesame protein/peptides mainly concentrate upon antioxidant activity and inhibitory activity against angiotensin-converting enzyme (ACE).

Oxidative stress plays an important role in arteriosclerosis, cardiovascular diseases, diabetes, cancer, traumatic brain injury, and other chronic diseases [48], and it is usually caused by the imbalanced metabolism of various reactive oxygen species (ROS), reactive nitrogen species (RNS), and other free radicals (R·) in organisms. Excessive oxidative stress can cause cell death, malignant proliferation, and canceration in the body. A diet rich in plant-based foods is associated with significantly decreased damage caused by antioxidants [49]. Proteins/peptides such as corn peptides and bean peptides have been reported to have antioxidant activity [50].

Table 1 presents the antioxidant peptides from sesame protein and their characteristics and activities. Sesame protein can be hydrolyzed to bioactive peptides inhibiting lipid peroxidation [51]. Interestingly, the antioxidant activities of all sesame cultivars differ; black sesame cultivars demonstrate higher values than white ones [52]. Compared with the standard alkali extraction of sesame protein, the enzyme-promoted method and the ultrasonic-assisted extraction method increase antioxidant capacity [17]. Chocolates prepared from enzymatic sesame meal protein exhibit high antioxidative activities, and they could be an alternative to chocolate confectionery products [53].

Sesame peptide also has inhibitory activity on ACE. It has been reported that the yield and inhibitory ACE activity of sesame peptide is 77.88% and 88.10%, respectively, after two-step hydrolysis with alcalase and flavourzyme [57]. It has been reported that 1 k Da peptide is the most active inhibitor of ACE; it exhibits an inhibitory ACE activity of 81%. Six ACE-inhibitory peptides were isolated and identified from SPP, among which the representative peptides are Leu-Val-Tyr, Leu-Gln-Pro, and Leu-Lys-Tyr [14]. Moreover, the activity of sesame peptides is also related to the proteases used for enzymatic hydrolysis, and the hydrolysate obtained from the enzymatic hydrolysis of sesame meal via alkaline protease displays the highest inhibitory activity against ACE [58]. Most oilseed hydrolysates such as sesame protein hydrolysates have the same ACE inhibition capacity as dairy protein [59], and thus they may be suitable substitutes for dairy protein. These results suggest that sesame peptide could be a beneficial ingredient in the prevention and treatment of hypertension and its related diseases, and that sesame peptide would be a good source for the development of functional foods.

In addition to the above-mentioned antioxidant activity and inhibitory activity against ACE, sesame protein also has good antibacterial activity. A novel antimicrobial protein (SiAMP2) belonging to the 2S albumin family has been isolated from sesame [60]. It has been confirmed that sesame contains antibacterial bioactive substances, supporting the hypothesis that plant storage proteins might play a role in pathogen prevention. Studies have evaluated the potential of antimicrobial peptide precursors from several oilseeds. The antimicrobial activities of six proteases have been analyzed, among which trypsin is the most effective in lyzing proteins, and three, four, two, and two antimicrobial peptides were obtained from soybean, peanut, sesame, and sunflower seeds, respectively [61]. In fact, sesame contains an antimicrobial peptide with a molecular mass of about 5.8 k Da, and this peptide exhibits antibacterial activity against *Klebsiella*, which causes human urinary tract infections [62]. Sesame protein hydrolysates have no inhibitory effect on *Salmonella* and *Escherichia coli*, but they show a significant inhibitory effect on *Staphylococcus aureus* [63]. The antibacterial activity of sesame protein provides a theoretical basis for the further development of antimicrobial peptides from oilseed protein. Sesame oil with high nutritional and therapeutic values exhibits diversified biological activities such as antimicrobial activity [64]. For example, sesame oil is a potentially useful natural additive to fresh meat products for improving its microbial quality and extending its shelf life during cold storage [65].

So far, there have been few reports on the bioactivities of sesame protein and sesame peptide. Therefore, it is necessary to conduct further research on the bioactivity of sesame meal protein and peptide for the high-value utilization of sesame byproducts.

### 3.2. Sesame Lignans

#### 3.2.1. Biological Activities of Sesamin

Sesamin, a significant lignan compound isolated from sesame, is well known for its antioxidant, anti-inflammatory, and tissue growth promotion properties.

**Figure 4:**
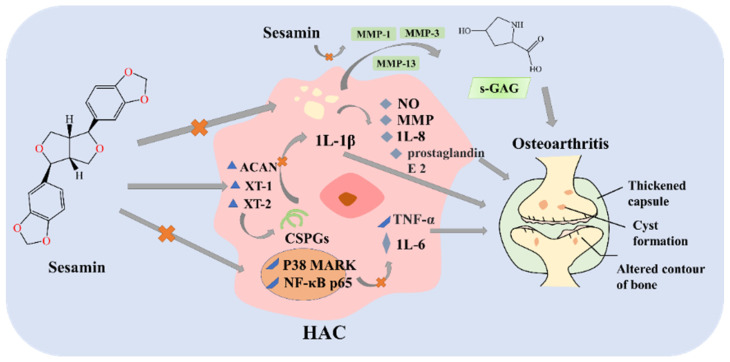
Schematic diagram of anti-inflammation by sesamin. HAC, human articular chondrocytes; GAGs, glycosaminoglycans; CSPGs, chondroitin sulfate proteoglycans; ACAN, XT-1, XT-2, chondroitin sulfate proteoglycan (CSPGs) synthesis genes; MMP, matrix metalloproteinase; IL-8, IL-6, prostaglandin E 2 and nitric oxide (NO); TNF-α, tumor necrosis factor-alpha; IL-1, interleukin-1.

Some studies have investigated the anti-inflammatory effects of sesamin on proteoglycan production in 3D chondrocyte culture. The results have demonstrated that sesamin enhances the synthesis of chondroitin sulfate proteoglycans (CSPGs), inhibits the expression of interleukin-1β (IL-1β), and alleviates IL-1β-induced inflammation in human chondrocytes (Figure 4) [66]. Another study [67] provided evidence that sesamin can attenuate mast-cell-mediated inflammatory cytokine release by suppressing p38 MAPK and NF-κB activation. Sesamin can reduce inflammatory mediators, thus relieving clinical symptoms and pathological changes caused by inflammatory impairment in patients with rheumatoid arthritis [68]. Therefore, sesamin can effectively suppress the pathological processes in an osteoarthritis model [69] to alleviate cardiac hypertrophy, inhibit fibrosis, and attenuate the inflammatory response [70]. 

Sesamin is a potential food supplement promoting bone health, and it benefits the differentiation of osteoblast progenitors towards functional osteoblasts (bone-forming cells). Sesamin coupled with osteogenic factor can significantly increase calcium deposition. During sesamin treatment, the reduction in absorption pit area and collagen release in bone slices indicate the inhibition of osteoclast differentiation [71]. 

Currently, sesamin has been proven to have positive effects on cardiovascular health. A recent meta-analysis indicated that intake of sesamin can decrease blood pressure. The antihypertensive effect of sesamin may be related to the sesamin-mediated inhibition of CYP4F2-catalyzed 20-hydroxyl eicosaenoic acid production [72]. Sesamin can also effectively decrease serum and liver fat levels [73]. Sesamin can increase the fatty acid oxidation rate of mitochondria and peroxisome in the liver of experimental animals [74]. Sesamin can also significantly improve the activity and gene expression of liver fatty acid oxidase, and it has a certain effect on blood lipid decrease [75]. The synergistic effect of soy phospholipid and sesamin can also improve liver fatty acid metabolism [74]. Sesamin is a promising natural compound for the treatment of hypercholesterolemia and the prevention of atherosclerosis as it reduces serum cholesterol level and LDL-C level (a risk factor for atherosclerosis), respectively, in humans. Studies have shown that the simultaneous intake of sesame and alpha-tocopherol can synergically reduce blood cholesterol concentration in rats fed a high-cholesterol diet by increasing biliary cholesterol excretion and reducing ApoB secretion into the blood [76]. Therefore, sesamin is a promising natural cholesterol-lowering healthcare product [77].

Moreover, sesamin can act as a potential therapeutic medicine for vessel-injury-related diseases [78] and exhibit certain anticancer activity. On the one hand, sesamin can effectively inhibit the growth and migration activity of endothelial cells. It can also suppress the expression of vascular endothelial growth factor (VEGF) in lung adenocarcinoma CL1–5 cells, therefore inhibiting the angiogenic activity of CL1–5 cells. On the other hand, sesamin can effectively inhibit macrophage-enhanced breast cancer cell activity via the inhibition of VEGF and matrix metalloproteinase (MMP-9) [79].

Interestingly, sesamin might have therapeutic potential in preventing and treating asthma and attenuating chronic mild stress-induced depressive-like symptoms. Sesamin can suppress the expression of lipopolysaccharide (LPS)-induced macrophage-derived chemokines (MDCs) via the endoplasmic reticulum (ER), the PPAR-alpha, the MAPK-p38 pathway, the NFκB-p65 pathway, and epigenetic regulation [80]. A recent study [81] indicated that sesamin attenuates chronic, mild stress-induced anxiety disorder symptoms by modulating dopamine, norepinephrine, serotonin, and corticosterone levels, as well as c-Fos expression.

#### 3.2.2. Biological Activities of Sesamolin

Sesamolin, one of the major sesame lignan compounds, possesses antioxidant, neuroprotective, and anticancer activities. Sesamolin reduces susceptibility to oxidative stress, enhances the oxidative stability of oil, and has antioxidant ability. Sesamolin can effectively inhibit the high-temperature-induced lipid peroxidation of soybean oil and enhance antioxidant ability with increasing amounts of sesamolin [82]. The antioxidant effect of sesamolin is comparable to that of butylated hydroxytoluene (BHT) at the same concentration, and it is superior to that of vitamin E. The antioxidant activity of the combination of sesamolin and phosphoric acid is significantly better than that of sesamolin alone. However, because sesamolin does not contain phenolic hydroxyl, its lipid antioxidant effect is not as good as that of sesamol, tert-butylhydroquinone (TBHQ), and other antioxidants [83]. Under the action of phosphoric acid, sesamolin is hydrolyzed into sesamol or partially rearranged into sesaminol, and both sesamol and sesaminol exhibit high antioxidant activity [84].

**Figure 5:**
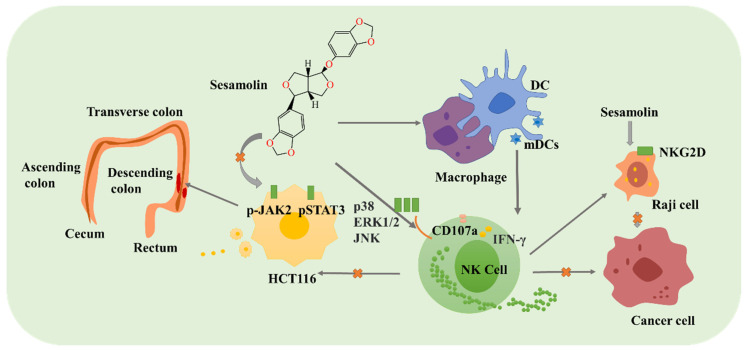
Schematic diagram of anticancer activity of sesamolin. Sesamolin regulates the anticancer mechanism of NK cells and Raji cells. HCT116, colorectal cancer cells; STAT3, transcription 3; DC, dendritic cells; NK cell, natural killer cells. Raji is a stable human cell line derived from the B-lymphocytes of a male Burkitt’s lymphoma patient.

Sesamolin has certain anticancer activity (Figure 5). Sesamolin can create an optimal environment for natural killer (NK) cells to kill cancer cells. Sesamolin activates NK cells by regulating the differentiation and activation of dendritic cells (DCs), thereby increasing the killing and migration activity of NK cells [85]. Sesamolin can increase the expression level of NKG2D ligands on Raji cells, exhibiting anticancer activity [86]. Sesamolin inhibits the proliferation and apoptosis of human colorectal cancer (HCT116) cells by suppressing the JAK2/STAT3 signaling pathways [87]. Sesamolin has the potential to be developed as a secure and effective treatment ingredient for colorectal cancer.

Sesamolin also has the dual role of blocking the generation of melanogenesis-related enzymes and inhibiting the enzymatic reaction of tyrosinase, exhibiting an anti-melanogenesis property in melanoma cell lines, and it is expected to be developed into a melanogenesis inhibitor [88]. Sesamolin affects genes related to angiogenesis and erythropoiesis [89], and sesarin (a type of sesamolin) can be used to develop drugs for treating Alzheimer’s disease [90]. 

#### 3.2.3. Biological Activities of Sesamol

Firstly, the most important biological activity of sesamol is its antioxidant activity. A previous study found that the antioxidant activity of sesamol is significantly higher than that of sesamin, sesamolin, or alpha-tocopherol [91], and sesamol plays an antioxidant role in the photooxidation process of oil as a singlet oxygen scavenger. Therefore, sesamol can be applied to anti-melanin production and skin protection [92], and it can reduce the melanin index and melanin content of the skin [93]. Sesamol might protect gastric mucosa against diclofenac (DLF)-induced injury by inhibiting hydroxyl-radical-associated lipid peroxidation.

Secondly, sesamol has been reported to ameliorate obesity by regulating lipid metabolism [94]. Sesamol treatment can alleviate ameliorate hepatic steatosis by inhibiting lipid accumulation and oxidative stress, suggesting that sesamol can be used as functional food ingredient for nutrition healthcare in the future [95]. 

Thirdly, sesamol has a protective effect on various central nervous system diseases, and the possible mechanisms of the neuroprotective effects of sesamol might be ApoE-dependent. The beneficial effects of sesamol on gut microbiota/metabolites could promote neurodegenerative disease treatment [96]. Additionally, sesamol shows potential as a therapeutic agent for improving asthma [97]. Sesamol exhibits antiangiogenic effects to prevent platelet activation, which is relevant to a variety of acute thrombotic events and coronary heart diseases [98].

Sesamol prevents the conversion of inactive inflammatory oxygenase (LOX) (Fe^2+^) into active LOX (Fe^3+^) by arresting the oxidation state of iron, prolonging the lag phase, and scavenging hydroperoxides [99]. Sesamol can effectively ameliorate DSS-induced colitis by promoting gut microecology [100]. The inhibitory effect of sesamol on lipoxygenase suggests its potential anti-inflammatory activity.

## 4. Comprehensive Utilizations of Sesame Protein and Sesame Lignans

Sesame meal is a discarded material in the oil pressing industry, and it contains a great deal of chemical compounds such as protein, minerals, dietary fiber, and vitamins [101]. The protein in sesame meal obtained by traditional methods is dramatically denatured; sesame meal is frequently used as animal feed, resulting in serious waste of protein resources [102]. How to take advantage of waste from food manufacture effectively and render it into valuable products and commodities remains to be further investigated. 

### 4.1. Utilization of Sesame Protein

The addition of processed sesame meal [103] decreases feed costs, and the processing can also ameliorate the crude protein and antioxidant content, enriching the mineral content of the original sesame meal. For example, defatting decreases phytic acid. Numerous studies have compared the effects of the addition of soybean meal and flaxseed meal to a fish diet with those of the addition of white sesame meal and black sesame meal. The results show that supplementation with sesame meal into the diet results in the high digestibility of protein and fat, significantly improving fish growth performance. The growth efficiency and feed utilization efficiency of rohu (fish farmed in southern Asia) fed on a fermented sesame meal diet are better than those of rohu fed on an oilseed meal diet [104], which might be because regular oilseed meal is short of methionine and lysine. 

Sesame protein, a plant-derived protein, is a cost-effective protein source, and it possesses great reserve force as a functional food ingredient. Sesame protein is regarded as a replenishment for the human diet [5]. Sesame protein and its hydrolysates can serve as additives in baked food or beverages as well as dietary supplements [105]. Sesame protein can be added during the manufacturing process of wheat bread, which not only improves the protein content and its absorption by humans, but also enriches the flavor of the bread [41].

### 4.2. Sesame Lignans

Sesame lignans mainly include sesamin, sesamolin, and sesamol. There have been few reports on the application of sesamin in the food industry. The prototype of osteogenic tissue engineering is prepared from polyhexene and sesamin via the electrospinning technique, and this prototype can be applied to biomedicine [106]. Based on the fact that sesamin can improve the activity of alcohol dehydrogenase, sesamin microcapsules are prepared via the agglomeration method so as to promote the commercial application of sesamin [107]. The extent, rate, and variability of the oral administration absorption of sesamin could be controlled using different types of lipid-based formulations (LBFs), indicating the possibility of developing better oral sesamin supplement products using appropriate formulations [108]. Solid acid catalysts are screened and applied to promote the conversion of sesamin into asarinin in sesame oil to improve the nutritional value of sesame oil [109]. Sesamin can be used for the development of skin anti-aging exploitation [110]. Sesamin can also be used as an available dietary supplement to improve blood pressure and lipids, and further as a healthcare product to prevent cardiovascular disease [111]. Sesamin can be applied as a pesticide in the field to effectively protect fruit from damage and scratches by termites [112].

Sesamolin provides prospects for practical applications such as oil–food processing, and it is used as an inartificial antioxidant due to its antioxidant effect [82]. For instance, the addition of sesamolin prolongs the oxidation induction time of soybean oil and delays the time of decrease in total unsaturated fatty acid content in soybean oil [113]. Sesamolin can also be converted into more active substances through technical means, such as conversion into sesaminol via cation exchange resin [114]. Sesaminol, a potential natural antioxidant, can be used as a food additive or medicine, but it is only a trace compound, and it can be isolated from sesamolin under proper and specific conditions. The cation exchange resin can effectively catalyze the conversion of sesamolin into sesaminol.

Sesamol, a natural antioxidant in edible oil, is lost in the process of evaporation or conversion during heating [115]. For the sake of improving the stability and water solubility of sesamol, β-cyclodextrin is used to embed sesamol, which broadens the application of sesamol in the food and medicine industries [116]. It is also possible to increase the antioxidant activity of sesamol at frying temperature by adding additives to reduce its volatility [117]. In terms of food processing, sesamol can enhance the oxidative stability of organogels and lipids to prolong the shelf life of meat products [118]. Additionally, sesamol can also suppress the generation of glycidyl ester, a food processing pollutant widely found in edible oil that mainly inhibits the hydrolysis and oxidation of oil [119]. Sesamol is a potential nutritional supplement to prevent the memory loss associated with an unhealthy diet [120].

Currently, the industrial application of sesame is limited by the production of sesame oil and sesame paste [121]. Lignans can be used as natural antioxidants, exhibiting broad prospects for development. 

## 5. Conclusions and Prospects

This review summarizes the composition, functions, biological activities, and comprehensive utilizations of sesame protein and sesame lignans (sesamin, sesamolin, and sesamol). These bioactive substances in sesame meal, the byproducts of sesame oil extraction, have promising development prospects and application potential. Sesame protein added to feed can be used as a substitute for nutritional sources, exhibiting antioxidant, antibacterial, and other activities. Sesame lignans can be used as nutritional components in healthcare products, presenting antioxidant, anti-inflammatory, and anticancer activities.

Future studies should be strengthened in the following directions. Firstly, the healthcare functions of sesame lignans and lignan-based healthcare products need to be further investigated and developed, respectively. The possibility and dose effects of sesame lignans in the development of healthcare food and anti-aging drugs should be further explored. Secondly, to utilize the high antioxidant activity of sesamol sufficiently, it is necessary to explore the mutual conversion mechanism of the three sesame lignans (sesamin, sesamolin, and sesamol). Thirdly, at present, the comprehensive utilization rate of sesame protein is low, especially in the human diet. This might be mainly due to the lack of research on the functional and biological activities of sesame protein, which limits the industrialized production and utilization of sesame protein. Future studies should pay more attention to the comprehensive utilization of industrial sesame byproducts, especially sesame protein as an important protein source, so as to avoid sesame resource waste.

## Figures and Tables

**Table 1:** Antioxidant peptides extracted from sesame proteins.

Source of Peptide	Detection Technology	Preparation	Activity	References
Defatted sesame meal		Bromelain flavourzyme	Hydroxyl and DPPH radical-scavenging activity	[54]
Sesame protein	Ultrafiltration and preparative HPLC	Dual-enzyme system comprised alcalase and trypsin	Nano liquid chromatography electrospray ionization–tandem mass spectrometry (Nano-LC-ESI-MS/MS)-CoMFA; DPPH and ABTS radical-scavenging activity; IC50	[51]
Sesame seed meal		Alkalase enzyme	DPPH free radical scavenging activity	[53]
Defatted sesame meal	Membrane ultrafiltration	Consecutive additions of pepsin and pancreatin	Radical scavenging and metal ion chelation	[14]
Sesame bran	Standard alkaline method	Viscozyme L, alcalase, ultrasound and ultrasound-assisted enzymatic extractions	DPPH and ABTS radical-scavenging activity	[17]
Defatted sesame meal		Pepsin, trypsin, chymotrypsin	PPH, ABTS and FRAP free radical	[55]
Sesame 11S protein		Alcalase and tripsin are mixed in an enzyme activity ratio of 1:1	DPPH and ABTS free radical scavenging rate	[15]
Sesame seed meal	Ultrasound-assisted technology	Broken; centrifugal	DPPH free radical and hydroxyl free radical	[56]

## Data Availability

The data presented in this study are available on request from the corresponding author.
